# A novel intra-ventricular assist device enhances cardiac performance in normal and acutely failing isolated porcine hearts

**DOI:** 10.1177/03913988211003912

**Published:** 2021-04-05

**Authors:** Daniël IM van Dort, Jos Thannhauser, Wim J Morshuis, Guillaume SC Geuzebroek, Dirk J Duncker

**Affiliations:** 1 Department of Cardiothoracic Surgery, Radboudumc, Nijmegen, The Netherlands; 2 Department of Cardiology, Radboudumc, Nijmegen, The Netherlands; 3 Department of Experimental Cardiology, Erasmus MC, Rotterdam, The Netherlands

**Keywords:** Blood pump design, blood pump balloon, experimental methods, mechanical support, cardiac assist and artificial heart, bioengineering

## Abstract

**Background:**

We recently demonstrated that a novel intra-ventricular membrane pump (IVMP) was able to increase the pump function of isolated beating porcine hearts. In follow-up, we now investigated the impact of the IVMP on myocardial oxygen consumption and total mechanical efficiency (TME) and assessed the effect of IVMP-support in acutely failing hearts.

**Methods:**

In 10 ex vivo beating porcine hearts, we studied hemodynamic parameters, as well as arterial and coronary venous oxygen content. We assessed cardiac power (CP), myocardial oxygen consumption (MVO_2_), and TME (CP divided by MVO_2_) under baseline conditions and during IVMP-support. Additionally, five isolated hearts were subjected to global hypoxia to investigate the effects of IVMP-support on CP under conditions of acute heart failure.

**Results:**

Under physiological conditions, baseline CP was 0.36 ± 0.10 W, which increased to 0.65 ± 0.16 W during IVMP-support (increase of 85% ± 24, *p* < 0.001). This was accompanied by an increase in MVO2 from 18.6 ± 6.2 ml/min at baseline, to 22.3 ± 5.0 ml/min during IVMP-support (+26 ± 31%, *p* = 0.005). As a result, TME (%) increased from 5.9 ± 1.2 to 8.8 ± 1.8 (50 ± 22% increase, *p* < 0.001). Acute hypoxia-induced cardiac pump failure reduced CP by 35 ± 6%, which was fully restored to baseline levels during IVMP-support in all hearts.

**Conclusion:**

IVMP-support improved mechanical efficiency under physiological conditions, as the marked increase in cardiac performance only resulted in a modest increase in oxygen consumption. Moreover, the IVMP rapidly restored cardiac performance under conditions of acute pump failure. These observations warrant further study, to evaluate the effects of IVMP-support in *in vivo* animal models of acute cardiac pump failure.

## Introduction

Cardiogenic shock, most often caused by acute myocardial infarction, is a medical emergency that requires immediate treatment to prevent multi-organ failure or even death.^1^ To improve outcomes, various mechanical circulatory support (MCS) devices have been developed. Apart from improving forward flow, the available devices reduce the cardiac workload, and consequently lower oxygen consumption, thereby improving cardiac efficiency.^2,3^ Disappointingly, these devices have not shown effectiveness in terms of patient survival.^4^

We have recently developed a novel cardiac assist device: the intra ventricular membrane pump (IVMP).^5^ As opposed to currently available assist devices, which are mainly based on passive or continuous unloading, the IVMP is designed to increase native stroke volume in a co-pulsatile manner. It consists of a dynamic volume that provides a 20 ml volume controlled displacement from the left ventricular (LV) apex toward the outflow track, through inflation of the device during systole, and deflation during diastole, as described previously.^5^ In a recent proof-of-concept study, we demonstrated the ability of the IVMP to augment stroke volume, cardiac output (CO), and mean aortic pressure (MAP) in an ex vivo beating healthy porcine heart model.

One potential concern of the IVMP and other intraventricular volume based devices,^6–9^ is that an increase in aortic- and left ventricular pressure increases LV afterload and thereby myocardial oxygen consumption (MVO_2_),^10^ potentially attenuating the IVMP-induced increase in CO. In our initial proof-of-concept study, MVO_2_ was not assessed. Therefore, the present study was designed to investigate the effect of IVMP-support on MVO_2_ and to determine whether IVMP-support alters the total mechanical efficiency (TME).^11^ In addition, we investigated the effect of IVMP-support during acute cardiac pump failure produced by global hypoxia, to mimic cardiogenic shock in a controlled fashion.

## Methods

### Experimental set-up and measurements

**Figure 1.**
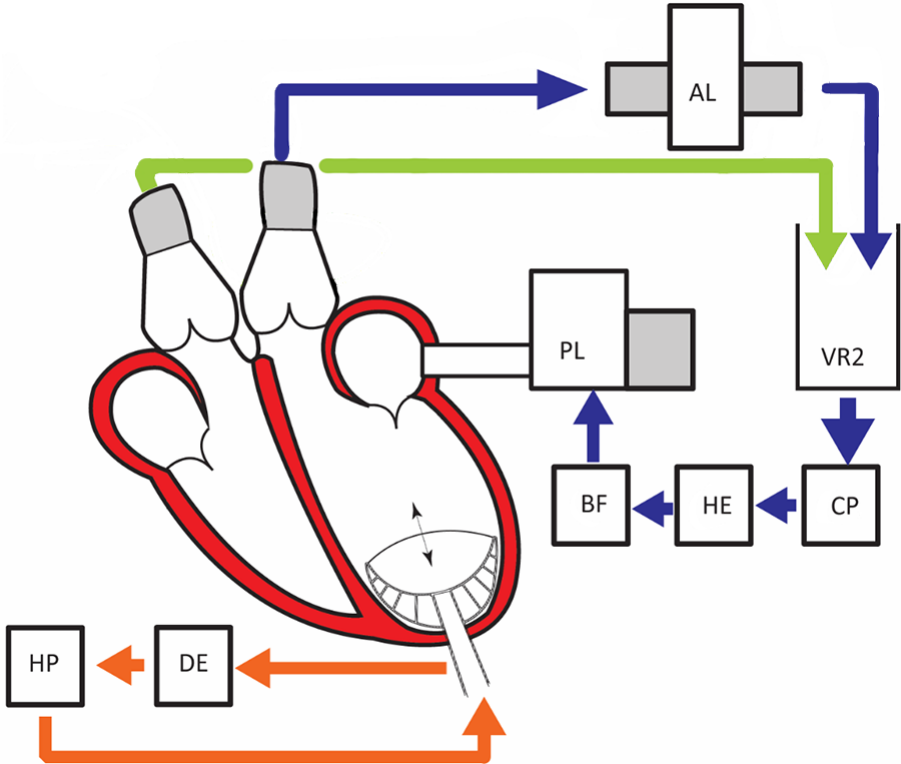
PhysioHeart-Platform, Blue represents the systemic circulation. The blood is pumped from the left ventricle to the after-load system (AL), Venous reservoir (VR2), continuous pump (CP), Heater and oxygenator (HE), Blood filter (BF), and ends back in the pre-load system. Green the pulmonary circulation, where coronary and venous saturation are measured continuously. Orange represents the device and pump control (HP helium pump; DE is delayed ECG signal).

All hearts were obtained from Dutch female Landrace hybrid pigs, which were slaughtered for human consumption and treated according to EC regulations 1774/2002 under supervision of the Dutch Government. The hearts were chosen for their similar size relative to human hearts (420 ± 30 g).^12^ They were arrested, isolated, and transported with a warm ischemia time of less than 5 min. The devices were implanted as described previously, and connected to the PhysioHeart platform (LifeTec Group, Eindhoven).^12^ As described previously,^5^ the platform consists of a pre- and afterload module, a venous reservoir, and a centrifugal pump (Figure 1). The interaction of the porcine heart and the platform has been engineered specifically to simulate the human cardiovascular system.

The IVMP is a volume-controlled pump, that displaces 20 ml blood from the apex to the outflow tract. The timing can be controlled relative to the cardiac cycle. Inflation was triggered at the start of systole, and deflation was timed when LVP was returned at diastolic pressure.

Continuous measurements were performed for the cardiac output (CO), coronary blood flow (CBF), aortic pressure (AoP), left ventricular pressure (LVP), and left atrial pressure (LAP). The cardiac power (CP) was calculated as described previously.^13^ Information on saturation and hemoglobin levels were continuously obtained and used for calculations of myocardial oxygen consumption (MVO2) and total mechanical efficiency (TME). The latter was defined as the ratio of CP and MVO2.^1^ A more detailed description of the parameter calculations can be found in the Supplemental Material.

### Experimental protocols

#### IVMP-support, physiological conditions

After preparation and connection to the platform, all 10 hearts were stabilized for 15 min, by means of Langendorff perfusion. After stabilization, the hearts were paced at 100–120 bpm, for reliable control of triggering of the support, after which baseline measurements were recorded. Subsequently, IVMP-support was initiated for a period of at least 3 min and measurements were performed during support. During support, the LAP was maintained stable with respect to the LAP-level of the baseline measurement.

#### IVMP-support, acute pump failure

A subset of five hearts was used to investigate hemodynamic responses to IVMP-support during hypoxia-induced acute pump failure. In these experiments, the arterial hemoglobin saturation was gradually reduced to 60% or less, resulting in an acute reduction in cardiac performance. In the PhysioHeart platform, heart failure is reflected by an increase of LAP, due to the control pump of the platform. The IVMP was initiated when there was a significant increase in LAP (defined as either three times the baseline value, or >20 mmHg). After a period of 3 min, support was terminated while data recording was continued until approximately 1 min after termination of support.

### Data recording and primary outcome measures

All pressures were monitored with pressure sensors (P10EZ-1; Becton Dickinson Medical, Franklin Lakes, USA). The pulsatile aortic flow was measured distally of the aortic valve annulus, immediately after the coronary ostia (MA28PAX; Transonic Systems Inc., Ithaca, USA) and the total CO was measured downstream the afterload module (HFM-09-1; HemoLab, Eindhoven, The Netherlands). The ECG-signal was acquired using leads directly on the heart (Streamline™ 6492; Medtronic, Minneapolis, USA). Venous oxygen saturation was measured directly after the right ventricle using an inline saturation measurement (CDI 500, Terumo, Shibuya, Tokyo, Japan), at a 10 Hz sample frequency. All other data were continuously recorded at 1000 Hz and stored for the duration of one scenario using LabVIEW software (V7.1, National Instruments, Austin, USA).

For the physiological experiments, 10-s segments were selected for analysis of the hemodynamic parameters during (1) a plateau phase at baseline and (2) under IVMP-support. For the acute heart failure experiments, 10-s segments were analyzed during (1) a plateau phase at baseline, (2) during crash, and (3) under IVMP-support. Moreover, we analyzed segments directly post-support and 1 min after support. All analyses were performed using MATLAB (Version R2018a, MathWorks, Natick, USA).

### Statistical analysis

Continuous variables were described as means ± standard deviations. Hemodynamic parameters between baseline, crash, and support were compared using a paired *t*-test. The differences in CP, MVO_2_, and TME expressed in percentages were used as primary outcome measures. A *p* < 0.05 was considered statistically significant. Analyses were performed with IBM SPSS Statistics 22.0 (IBM Corp, Armonk, NY, USA).

## Results

### Total mechanical efficiency experiments

#### Baseline measurements

**Figure 2.**
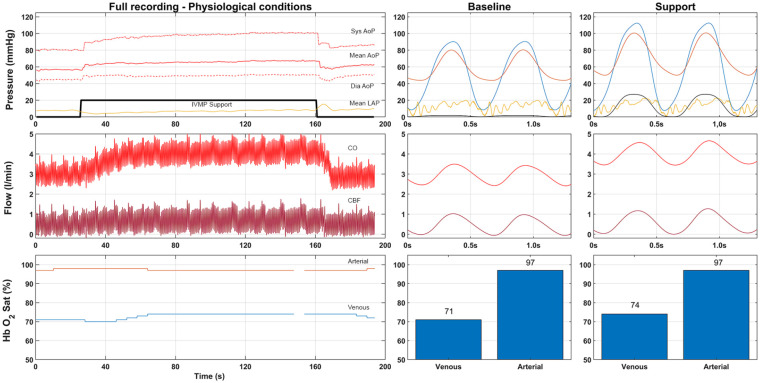
Example measurement of the total mechanical efficacy experiment. Upper part is pressure in mmHg (Red, Aortic pressure; Yellow, Left Atrial Pressure; Blue Left ventricular pressure; Black, pump pressure scaled by a factor 10). Flow is represented in the middle part (total cardiac output red, coronary flow dark red). Lower bar represents the saturation (Arterial saturation in red, venous in blue). Center and right side provide individual wave forms of supported and unsupported setting.

Implantation of the IVMP was successfully performed in all 10 hearts. A representative example of a measurement is shown in Figure 2. All baseline hemodynamic parameters are reported in Table 1. At baseline, the mean MAP was 57 ± 7 mmHg, the mean CO was 2.8 ± 0.6 l/min, and the mean LAP was 16 ± 5 mmHg. The CO waveform did not show any indication of aortic valve regurgitation during IVMP support.

**Table 1.** Results of the primary analyses. All 10 hearts were tested under normal oxygenated conditions at baseline and during support with the IVMP (intraventricular membrane pump).

	Baseline	IVMP support	Change	*p*-value
Hemodynamics				
Heart rate (bpm)	113 ± 6	113 ± 6	0%	0.8
Aortic pressure (mmHg)				
Mean	56.9 ± 6.5	72.8 ± 4.4	+29% ± 13	<0.001
Systolic	78.1 ± 6.5	100.4 ± 5.7	+29% ± 10	<0.001
Diastolic	40.1 ± 7.5	49.1 ± 5.0	+26% ± 19	<0.001
Left ventricular pressure (mmHg)*				
Systolic	75.7 ± 11.7	98.6 ± 10.8	+32% ± 13	<0.001
Diastolic	5.4 ± 5.6	1.7 ± 5.8	+17% ± 146	<0.001
Left atrial pressure (mmHg)	15.7 ± 4.8	14.8 ± 2.4	−3% ± 14	0.293
Cardiac output (l/min)	2.8 ± 0.6	4.0 ± 0.8	+43% ± 8	<0.001
Coronary blood flow (ml/min)	548 ± 247	795 ± 269	+56% ± 38	<0.001
Oxygen measures				
Saturation (%)				
Arterial	98 ± 0.01	98 ± .01	+0% ± 0	0.431
Venous	73 ± 0.06	77 ± .06	+5% ± 3	<0.001
Cardiac performance measures				
Cardiac power (W)	0.36 ± 0.10	0.65 ± 0.16	+85% ± 24	<0.001
MVO_2_ (ml/min)	18.6 ± 6.2	22.3 ± 5.0	+26% ± 31	0.005
Total mechanical efficiency (%)	5.9 ± 1.2	8.8 ± 1.8	+50% ± 22	<0.001

Values are reported as means (standard deviations).

* Left ventricular pressure information was only available in eight hearts.

#### Cardiac performance, baseline versus IVMP support

All measurements of cardiac performance during the physiological experiments are reported in Table 1. CP at baseline was 0.35 ± 0.10 W. During IVMP support this increased to 0.65 ± 0.16 W (+85%, *p* < 0.001). The MVO2 at baseline was 18.6 ± 6.2 ml/min, which significantly increased to 22.3 ± 5.0 ml/min (+26%, *p* = 0.005). As a result, the TME significantly increased by 50% (5.9 ± 1.2% at baseline to 8.8 ± 1.8% during IVMP support, *p* < 0.001).

### Experiments with hypoxia-induced acute heart failure

#### Baseline versus hypoxia

**Figure 3.**
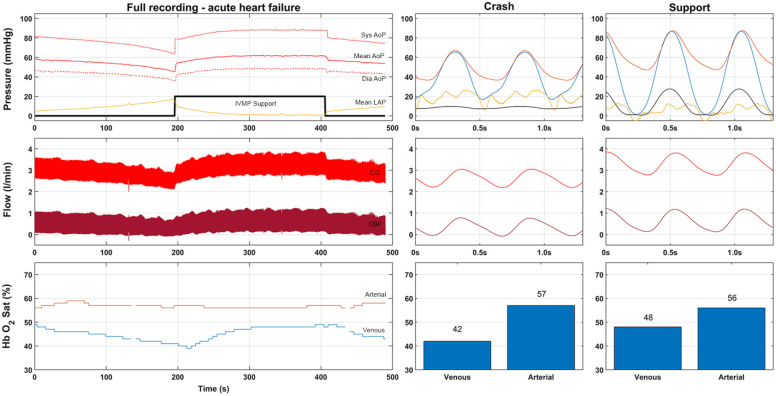
Example measurement of the pan hypoxia experiments. Upper part is pressure in mmHg (Red, Aortic pressure; Yellow, Left Atrial Pressure; Blue Left ventricular pressure; Black, pump pressure scaled by a factor 10). Flow is represented in the middle part (total cardiac output red, coronary flow dark red). Lower bar represents the saturation (Arterial saturation in red, venous in blue). Center and right side provide individual waveforms of crash and supported setting.

**Figure 4.**
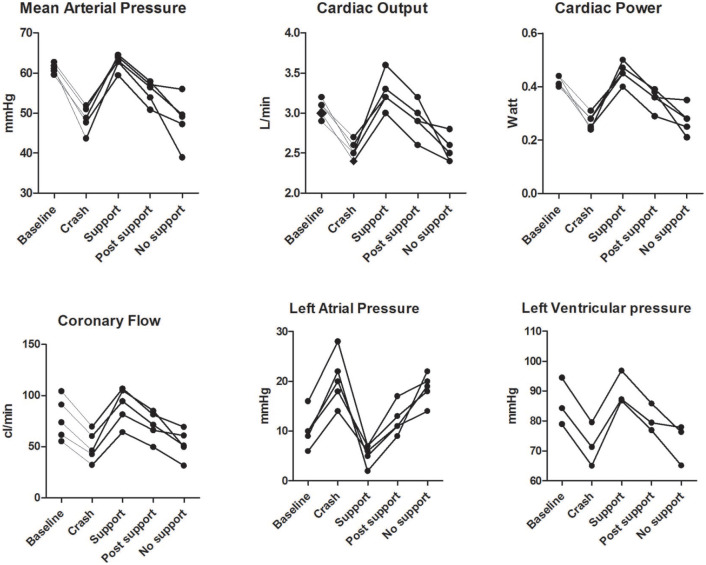
Individual measurements of exploratory crash experiments. Various hemodynamic parameters are plotted over time. Baseline is the initiation of reduced oxygenation, crash is the peak of heart failure under hypoxia, support is the plateau phase of IVMP support. Post support is measured directly after support and no support is at least 30 s after termination of support.

Figure 3 shows a representative example of a hypoxia induced heart failure experiment. All the hypoxia experiments are reported in Table 2 and Figure 4. We observed a gradual reduction in arterial saturation from 88 ± 17% to 56 ± 14%, which resulted in an increase in LAP (+200%) and a decrease in MAP (−18%) and CBF (−35%). Consequently, CP decreased from 0.42 ± 0.02 to 0.27 ± 0.03 W (−35%, *p* = 0.003).

**Table 2.** Hypoxia experiments. Effect of IVMP-support on hemodynamics and cardiac performance, after hypoxia-induced acute pump failure.

	Baseline	Crash	Change crash vs baseline	IVMP support	Change support vs crash
Hemodynamics					
Heart rate (bpm)	110 ± 6	109 ± 7	0 ± 0%	109 ± 7	0 ± 0%
Aortic pressure (mmHg)					
Mean	61.2 ± 1.2	48.6 ± 3.2	−21% ± 5^§^	62.7 ± 1.9	+29% ± 9^§^
Systolic	83.4 ± 3.3	68.6 ± 4.4	−18% ± 4^§^	86.9 ± 3.1	+27% ± 8^§^
Diastolic	43.0 ± 3.1	33.4 ± 4.3	−22% ± 6^§^	42.8 ± 3.9	+29% ± 10^§^
Left ventricular pressure (mmHg)*					
Systolic	85.9 ± 7.9	71.9 ± 7.3	−16% ± 1^§^	90.3 ± 5.6	+26% ± 7^§^
Diastolic	5.6 ± 1.2	17.2 ± 3.6	+308% ± 5^§^	−0.1 ± 2.5	−99% ± 13^§^
Left atrial pressure (mmHg)	10.4 ± 3.6	20.1 ± 5.0	+200% ± 34^§^	5.5 ± 2.1	−71% ± 13^§^
Cardiac output (l/min)	3.1 ± 0.1	2.5 ± 0.1	−18% ± 4^§^	3.3 ± 0.2	+29% ± 9^§^
Coronary blood flow (ml/min)	772 ± 204	502 ± 148	−35% ± 4^§^	904 ± 178	+86% ± 31^§^
Oxygen measures					
Saturation (%)					
Arterial	88 ± 17	56 ± 14	−33% ± 22^§^	51 ± 13	−8% ± 8^§^
Venous**	61 ± 19	46 ± 7	−22% ± 13	49 ± 1	+7% ± 12
Cardiac performance measures					
Cardiac power (W)	0.42 ± 0.02	.27 ± 0.03	−35% ± 7^§^	0.45 ± 0.04	+68% ± 24^§^
MVO_2_** (ml/min)	13.8 ± 9.6	10.8 ± 6.0	−13% ± 17	15.0 ± 10.8	+30% ± 27
Total mechanical efficiency** (%)	11.8 ± 8.5	9.0 ± 4.9	−18% ± 18	12.3 ± 9.1	+29% ± 32

Baseline measurements were performed at the initiation of pan hypoxic heart failure; crash is the peak of heart failure; support is measured in a plateau phase of the support.

* Data based on *n* = 3. **data based on *n* = 2. ^§^significant change (*p* < 0.05).

#### Hypoxia versus IVMP support

Under IVMP-support, we observed an immediate increase in systolic LVP (+18.4 mmHg, +26%), accompanied by an increase in CO and MAP (+0.8 l/min and +14 mmHg respectively, both +29%). Moreover, we observed a decrease in diastolic LVP from 17.0 ± 4.1 mmHg to 0.0 ± 2.5 mmHg, as well as a decrease in LAP from 20.1 ± 5.0 mmHg to 5.5 ± 2.1 mmHg (−14 mmHg, −71%). As a result, the cardiac power increased from 0.27 ± 0.03 W to 0.45 ± 0.04 W (+68%). Due to the low coronary venous oxygen saturations, data acquisition was not feasible during all experiments and therefore MVO2 and TME values could only be acquired in two out of the five hearts. In these hearts, MVO2 increased by 30% to 15.0 ± 10.8 ml/min, resulting in a 29% increase in TME to 12.3 ± 9.1%.

## Discussion

This study shows that co-pulsatile support by the IVMP significantly increases CP and TME under physiological conditions, as well as in acutely failing hearts. Importantly, the marked hypoxia-induced decrease in cardiac function was restored to baseline levels within a minute after initiation of IVMP-support. These promising results stimulate further development of a catheter-based IVMP to test in an in vivo setting.

### Total mechanical efficiency

In the first part of our study, we investigated the LV hemodynamic and metabolic responses to IVMP support. IVMP increased cardiac output and stroke volume by 45% and LV systolic pressure by 32%, resulting in an 85% increase in CP. These hemodynamic responses were accompanied by a modest 26% increase in MVO_2_ but yielding a significant 50% increase in TME. Since LAP—and thus LV diastolic dimension—was maintained constant during IVMP support in the physiological experiments, the increase in MVO_2_ was most likely the result of the increase in LV systolic pressure. The observed increase in MVO_2_ was particularly modest, considering that it has been previously been suggested that typical changes in CP produced by the heart itself are accompanied by quantitatively similar changes in MVO2, with minimal changes in TME.^14–16^ The observations in the present study suggest that a significant additional kinetic energy provided by the IVMP, only causes a mild increase in myocardial energy requirement.

### Hypoxia-induced acute heart failure

To explore the potential benefit of IVMP under conditions of cardiogenic shock, we investigated the effect of the IVMP in a model of acute heart failure. For this purpose, we used severe arterial hypoxia, since this produces a predictable and good degree of cardiac pump dysfunction and a lower chance of ventricular fibrillation, which is imminent when using focal ischemia for the reduction of pump function.^17^ We reduced the arterial hemoglobin saturation to approximately 60%, resulting in a progressive LV functional deterioration associated with a decrease in AoP and an increase in LAP. Upon initiation of IVMP-support, we observed immediate improvement of hemodynamic function (Figures 3 and 4, Table 2), and rapid return of LAP to—even lower than—baseline levels. Importantly, CO, LVSP, AoP, and CBF all showed a full recovery to—and above—baseline levels, showing that the additional kinetic energy provided by the pump has a net favorable influence on the acutely failing heart.

Interestingly, we observed a smaller IVMP-induced augmentation of CP in the hypoxia experiments in comparison with the physiological experiments (68% vs 85%). This difference is most likely related to differences in the preload setting in both conditions. We maintained LAP constant in the physiological experiments, thereby preventing lowering of LV preload^18^ (and thus the stroke volume and CO) by the IVMP-induced augmentation of stroke volume, which may have enhanced the increase in CP. In contrast, LAP was not controlled in the hypoxia experiments. This approach was chosen as it allowed investigation of the effects of hypoxia and subsequent IVMP support on LV hemodynamics, including LV filling pressures and congestion. As a potential result, LAP showed a marked increase during hypoxia, followed by a similar decrease during subsequent IVMP-support. The latter may have blunted the IVMP-induced increase in CO and CP as compared to the physiological experiments in which a decrease in LAP was prevented.^19^ Alternatively, it could be argued that differences in afterload contributed to the greater increase in CP in normoxia compared to hypoxia. However, all the other hemodynamic variables were comparable between normoxia and hypoxia conditions with even slightly lower aortic and LV systolic pressures in hypoxia conditions just prior to IVMP support, which would favor a larger increase in hypoxia conditions.

### Comparison with other synergetic devices

The positive effect of a synergetic pumping mechanism as we observed with the IVMP in the present study, has previously been reported for the Physiological Cardiac Assist Device (PCAD).^9^ This surgically implanted LVAD consists of a volume chamber (18 cc) and a single cannula, that is, connected to the apex. Such an external device fills during diastole and ejects during systole via the same cannula. Support by the PCAD resulted in a 0.17 W increase in CP.^20^ In our study, the IVMP showed a larger increase in CP (+0.29 W), but a similar increase in stroke volume (18 cc PCAD vs 20 cc IVMP). Also similar to our observations, PCAD-support resulted in an increase of CBF and oxygen content of the coronary sinus.^20^ The similarities are striking when considering that the PCAD was studied in an in vivo setting, while our study was performed in an ex vivo beating heart. More recently, the effect of pulsatile support with a long term LVAD on TME was tested in an ex vivo setting similar to the present study, essentially confirming our present findings of superior TME under pulsatile support.^21^ Thus, all three available studies indicate that synergetic pumping has a positive effect on the cardiac function and mechanical efficiency.

### Clinical setting and perspective

As stated above, different devices are available to provide circulatory support in the critical setting of cardiogenic shock, including the Impella and Intra-aortic balloon pump (IABP).^1,22^ These devices focus on unloading of the left ventricle. However, LV unloading may occur, at least partly, at the expense of the native CO which may explain preclinical studies reporting a limited improvement of CO under support with these devices.^23–25^ Similar studies stated that only a portion of the pump support translates into a CO-increase in the clinical setting.^26^ Where the Impella seems to be superior to the IABP in terms of added pumping power,^27^ both not to a satisfactory level.

The early initiated unloading, has been shown to reduce infarct size in animals,^28,29^ and was also observed to have a favorable outcome in terms of survival in a recent clinical study.^30^ Unfortunately, early support is not feasible in many cases, as the mean time-to-support is approximately 150 min in dedicated centers.^4^ The gap between ideal timing of unloading in the setting of cardiogenic shock and real-world challenges may explain the modest benefit of unloading on survival so far.^27,31^

The quick recovery of pump function and the sustained increase in CP during continued hypoxia as demonstrated in the present study, suggests a potential benefit of IVMP-support in the setting of acute cardiogenic shock. Hence, our results provide a stimulus for future studies to develop a catheter-based inflatable support device to be used in the clinical setting.

Co-pulsatile support is still in a conceptual phase. In this experimental setting we show promising data. Besides the lesion of implantation, we did not observe any other structural damage to the endocardium, there were no signs of aortic valve regurgitation. With regard to other possible comorbidities and complications, we recommend performing dedicated animal studies, with specific focus on the occurrence of arrhythmias and thrombus formation. Moreover, there are hurdles to overcome for the translation to the clinical setting. For the indication of cardiogenic shock, the device needs to be developed into a catheter-based device, and additional more in-depth investigation of anticipated anticoagulation is needed. Furthermore, the possible risks need to be identified and mitigated. Future studies are required to address these important translational questions.

### Limitations

There are several methodological aspects of the study that should be considered.

First, the prototype was implanted prior to baseline measurements, with a possible negative effect on cardiac pump function by the intraventricular placement of the IVMP. Furthermore, it was not possible to differentiate the work performed by the heart versus the work performed by this early-stage prototype. When subtracting the work performed by the device, the energetic profile of the heart alone may be less positive. However, our observation of rapid recovery under IVMP-support, suggests an overall beneficial effect of the device.

Second, during hypoxia, investigation of TME was not possible in three hearts in which the coronary venous hemoglobin oxygen saturations fell below the lower detection limit of the measurement equipment. As a consequence, we were able to obtain only two reliable measurements (Table 2, Figure 3), which demonstrated an average increase of 29% in TME. Moreover, the recovery in hemodynamic parameters strongly suggests that the IVMP provides overall positive synergetic support to the failing heart.

Third, for our study we used an isolated heart model, and we cannot exclude that the harvesting of the heart, the warm- and cold ischemia time and the cardioplegia may have influenced cardiac performance and TME. Moreover, there may have been some degree of myocardial stunning, also given the TME value at baseline of only 6% and the rather high LV filling pressures (LAP of 15 mmHg). However, this value of 6% is comparable to TME values (5%) observed in isolated beating porcine heart setting^21^ and in the range of values observed in ex vivo canine models.^32^

Lastly, to induce acute heart failure, we used global hypoxia. Although hypoxia is one component of ischemia, it is not identical to ischemia. Future studies should be aiming at studying the effects of IVMP during acute ischemia-induced cardiac pump failure in vivo with an advanced IVMP prototype.

## Conclusion

In the present study, performed in an ex vivo beating porcine heart model, IVMP-support resulted in a marked increase in cardiac power, accompanied by only a modest increase in myocardial oxygen consumption. These collective findings resulted in an increase in total mechanical efficiency. Interestingly, in the setting of hypoxia-induced acute cardiac pump failure, IVMP-support resulted in a quick and full restoration of cardiac performance. These results provide a rationale for the further development of a catheter-based IVMP for testing in in vivo animal models.

## Supplemental Material

sj-pdf-1-jao-10.1177_03913988211003912 – Supplemental material for A novel intra-ventricular assist device enhances cardiac performance in normal and acutely failing isolated porcine hearts Click here for additional data file. Supplemental material, sj-pdf-1-jao-10.1177_03913988211003912 for A novel intra-ventricular assist device enhances cardiac performance in normal and acutely failing isolated porcine hearts by Daniël IM van Dort, Jos Thannhauser, Wim J Morshuis, Guillaume SC Geuzebroek and Dirk J Duncker in The International Journal of Artificial Organs

sj-tif-2-jao-10.1177_03913988211003912 – Supplemental material for A novel intra-ventricular assist device enhances cardiac performance in normal and acutely failing isolated porcine hearts Click here for additional data file. Supplemental material, sj-tif-2-jao-10.1177_03913988211003912 for A novel intra-ventricular assist device enhances cardiac performance in normal and acutely failing isolated porcine hearts by Daniël IM van Dort, Jos Thannhauser, Wim J Morshuis, Guillaume SC Geuzebroek and Dirk J Duncker in The International Journal of Artificial Organs

sj-tif-3-jao-10.1177_03913988211003912 – Supplemental material for A novel intra-ventricular assist device enhances cardiac performance in normal and acutely failing isolated porcine hearts Click here for additional data file. Supplemental material, sj-tif-3-jao-10.1177_03913988211003912 for A novel intra-ventricular assist device enhances cardiac performance in normal and acutely failing isolated porcine hearts by Daniël IM van Dort, Jos Thannhauser, Wim J Morshuis, Guillaume SC Geuzebroek and Dirk J Duncker in The International Journal of Artificial Organs

sj-tif-4-jao-10.1177_03913988211003912 – Supplemental material for A novel intra-ventricular assist device enhances cardiac performance in normal and acutely failing isolated porcine hearts Click here for additional data file. Supplemental material, sj-tif-4-jao-10.1177_03913988211003912 for A novel intra-ventricular assist device enhances cardiac performance in normal and acutely failing isolated porcine hearts by Daniël IM van Dort, Jos Thannhauser, Wim J Morshuis, Guillaume SC Geuzebroek and Dirk J Duncker in The International Journal of Artificial Organs
