# Supplementary material for: A novel intra-ventricular assist device enhances cardiac performance in normal and acutely failing isolated porcine hearts
Source: Int J Artif Organs. 2021 Apr 5;45(4):388–96. doi: 10.1177/03913988211003912 (PMC8921884; doi:10.1177/03913988211003912)
Supplement: sj-pdf-1-jao-10.1177_03913988211003912 – Supplemental material for A novel intra-ventricular assist device enhances cardiac performance in normal and acutely failing isolated porcine hearts [file sj-pdf-1-jao-10.1177_03913988211003912.pdf]

Calculations

For the current study, we calculated the cardiac power (CP) using the cardiac output (CO) and mean arterial pressure (MAP), following:

$$\textit{Cardiac power (Watt)} = \left(\frac{COxMAP}{451}\right)$$

Oxygen consumption was calculated as a measure of energy usage. Here, we assume that all metabolic activity is aerobic, although in an in-vivo setting the metabolism may switch to anaerobic metabolism. O_2-content of the blood is given in µmol/ml and computed as:

$$O_2 \text{ content} = (0.621 \cdot \text{Hba (g/dL)} \cdot SO_2)$$

The PO_2 was neglected since it has only limited added value in the measurement. The oxygen consumption (MVO_2) was measured using the arterial and venous O_2-content and the coronary blood flow (CBF), following:

$$\textit{MVO}_2\ \textit{(mLO}_2\textit{/min)} = (\text{arterial } O_2 \text{ content - venous } O_2 \text{ content}) * \text{CBF (mL/min)}$$

The MVO_2 can be converted into energy (1ml O_2 = 20.2 Joule). For the efficiency we combined the two parameters, CP (W) and MVO_2 (J) following:

$$\text{Total mechanical efficiency (TME)} = CP / MVO_2$$
