# Supplementary figures and images for: A novel intra-ventricular assist device enhances cardiac performance in normal and acutely failing isolated porcine hearts
Source: Int J Artif Organs. 2021 Apr 5;45(4):388–96. doi: 10.1177/03913988211003912 (PMC8921884; doi:10.1177/03913988211003912)

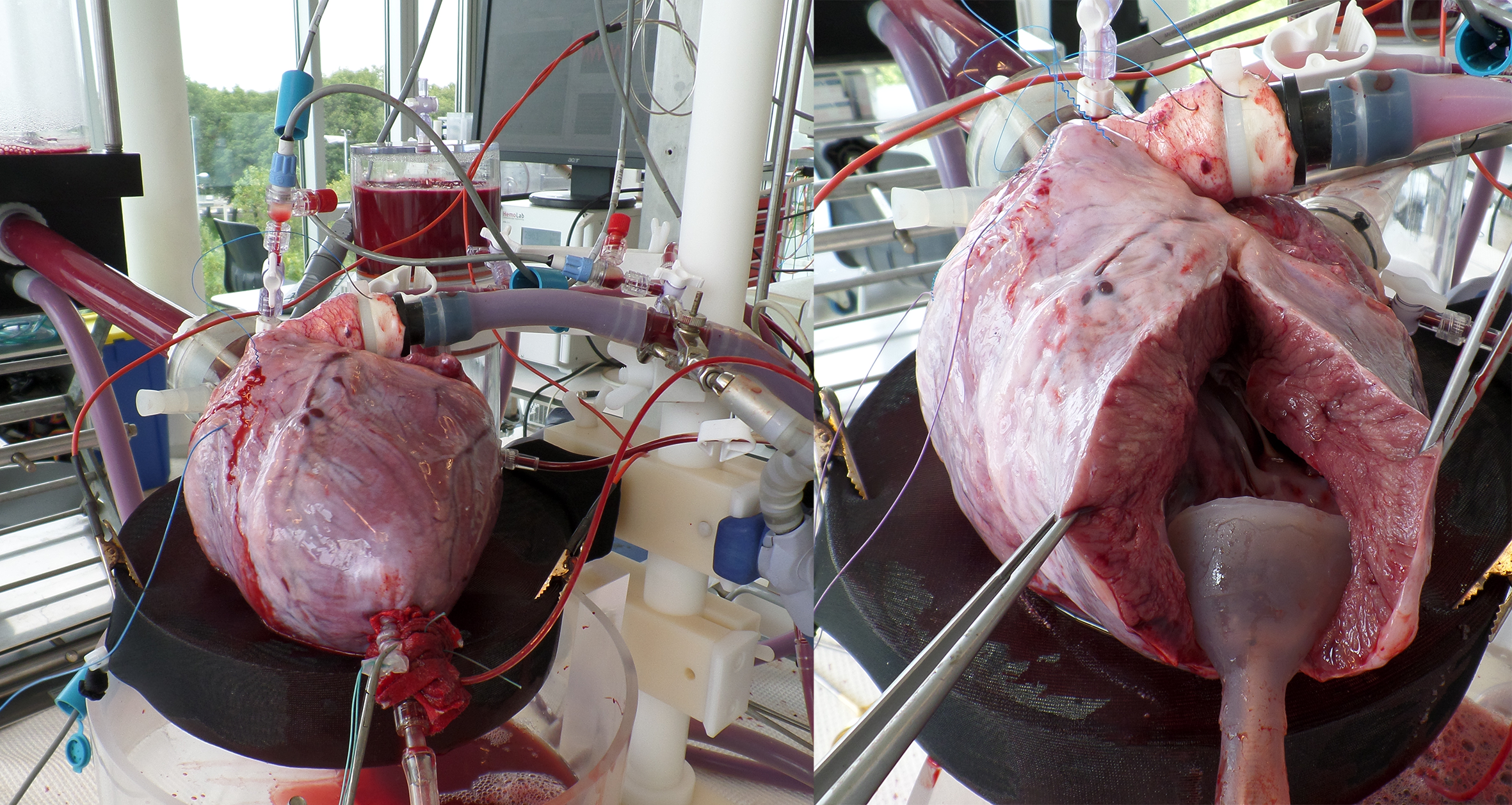

Supplement: sj-tif-2-jao-10.1177_03913988211003912 – Supplemental material for A novel intra-ventricular assist device enhances cardiac performance in normal and acutely failing isolated porcine hearts [file sj-tif-2-jao-10.1177_03913988211003912.tif]

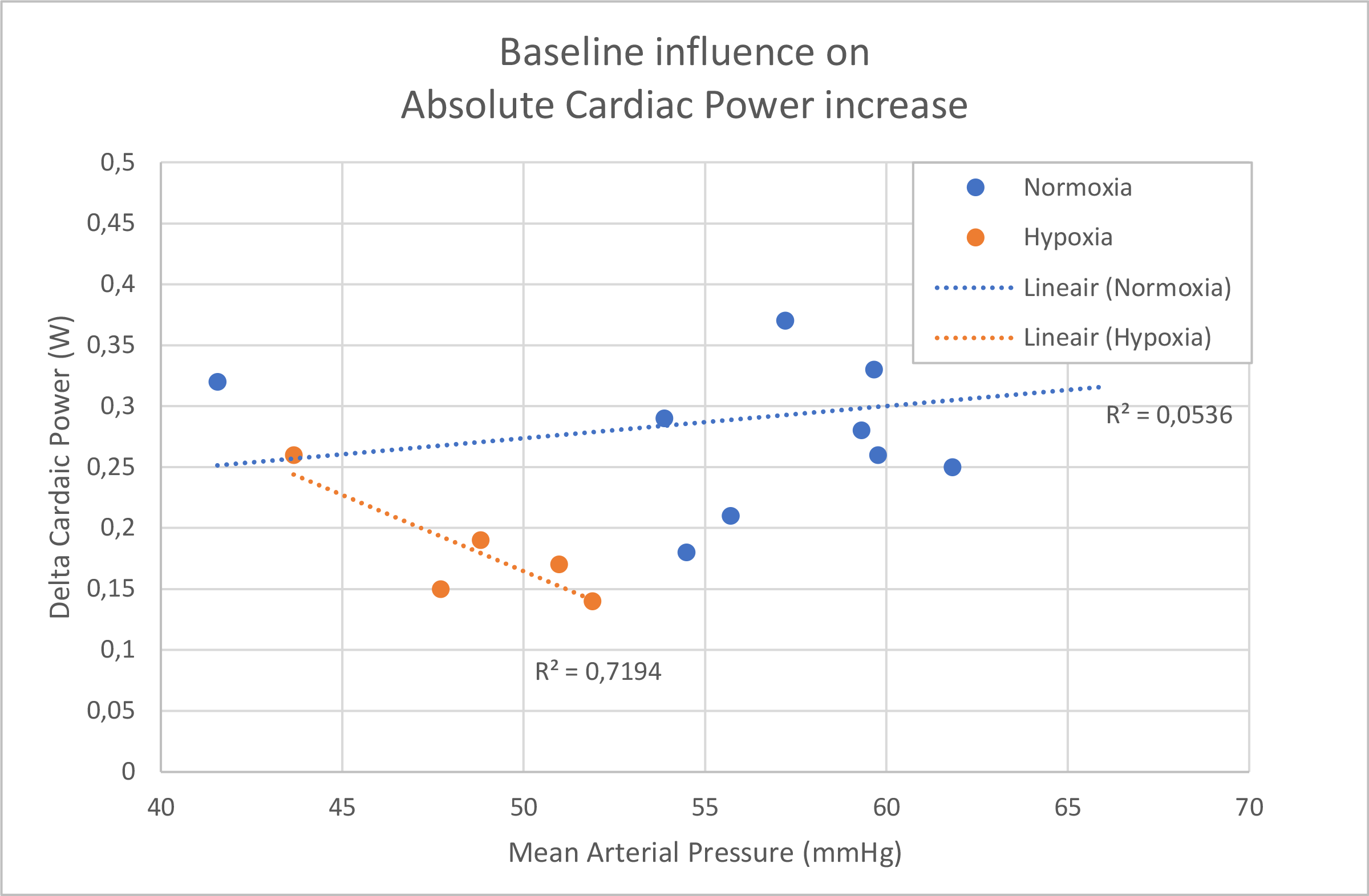

Supplement: sj-tif-3-jao-10.1177_03913988211003912 – Supplemental material for A novel intra-ventricular assist device enhances cardiac performance in normal and acutely failing isolated porcine hearts [file sj-tif-3-jao-10.1177_03913988211003912.tif]

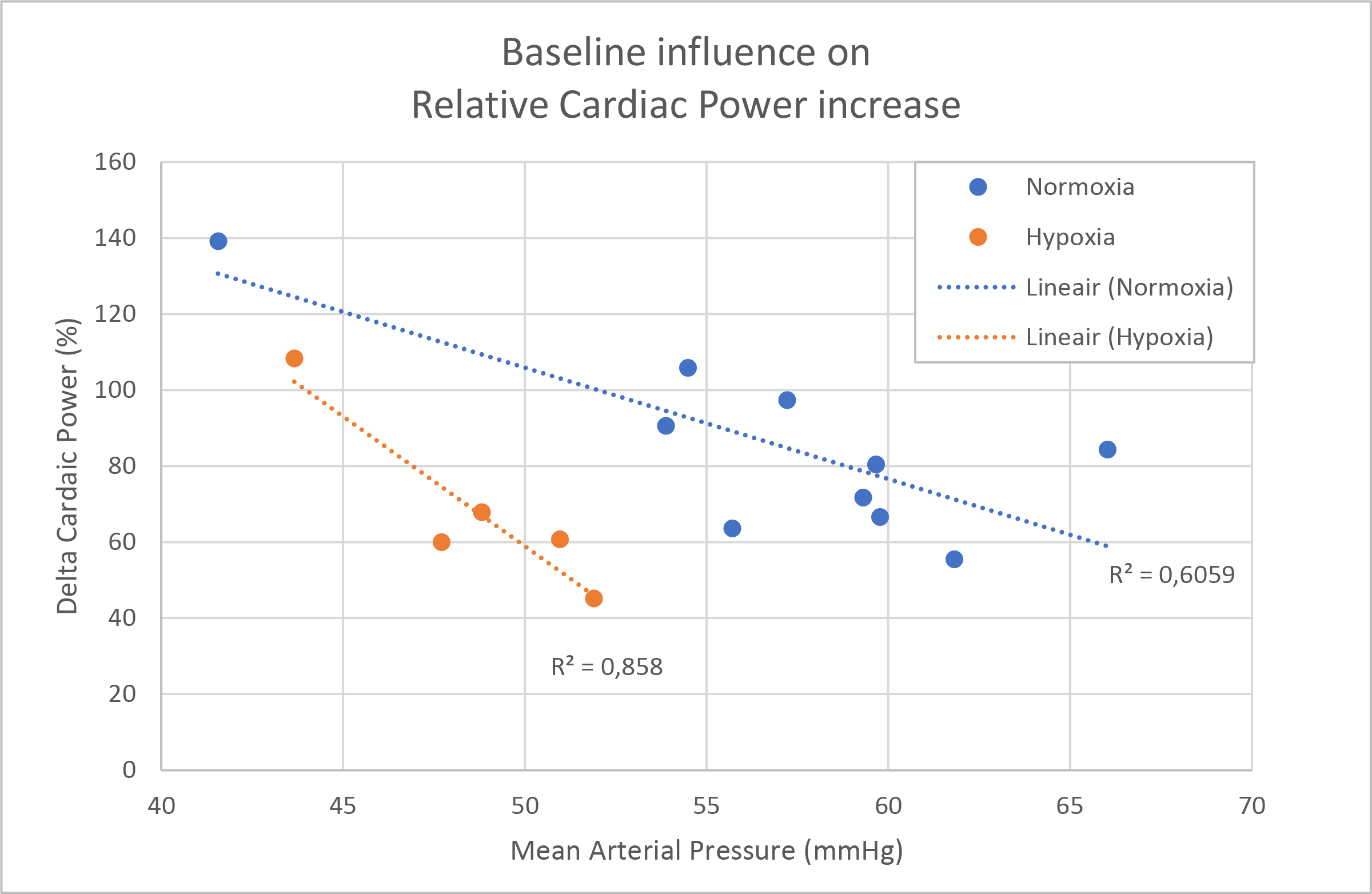

Supplement: sj-tif-4-jao-10.1177_03913988211003912 – Supplemental material for A novel intra-ventricular assist device enhances cardiac performance in normal and acutely failing isolated porcine hearts [file sj-tif-4-jao-10.1177_03913988211003912.tif]
